# Primary undifferentiated pleomorphic sarcoma of the breast in a young female: a case report

**DOI:** 10.1186/s12957-016-0947-9

**Published:** 2016-07-16

**Authors:** Banushree C. Srinivasamurthy, Ambedkar Raj Kulandaivelu, Kaushik Saha, Arpita Saha

**Affiliations:** Department of Pathology, All India Institute of Medical Sciences, Sijua Village, Dumduma, Bhubaneswar, Orissa-17 India; Department of Pathology, Chennai Medical College, Trichy, India; Present Address: Department of Pathology, Indira Gandhi Medical College, Puducherry, 605113 India

**Keywords:** Sarcoma, Undifferentiated, Breast, Immunohistochemistry, Diagnostic challenge

## Abstract

**Background:**

Undifferentiated pleomorphic sarcoma is a rare entity and requires extensive immunohistochemical markers to differentiate it from other tumors of the breast.

**Case presentation:**

We present a 29-year-old female with a left breast lump for 2 months. Initial diagnosis of malignant spindle cell tumor was done on core biopsy following which total mastectomy was done. After extensive sampling, on histology, highly pleomorphic spindle cells palisading the area of geographic necrosis with very high atypical mitosis were seen. As there was diagnostic difficulty, immunohistochemical antibody panel was used and diagnosis of undifferentiated pleomorphic sarcoma of the breast was made by exclusion.

**Conclusions:**

Core biopsy with immunostaining is possibly superior to FNA as an initial diagnostic modality for breast masses with atypical features.

## Background

Primary breast sarcomas are uncommon, histologically heterogenous non-epithelial malignancies that arise from the connective tissue within the breast [1]. Undifferentiated pleomorphic sarcoma constitutes less than 5 % of all sarcomas in adults and has been rarely seen in the breast and is defined as a group of pleomorphic, high-grade sarcomas in which any attempt to disclose their line of differentiation has failed [2, 3]. Most undifferentiated pleomorphic sarcomas have occurred in their sixth and seventh decades of life and very rarely in adolescents and adults [4]. Differentiating sarcoma subtypes based on molecular characteristics helps in differential treatment sensitivities and development of specifically targeted therapies in sarcomas especially in breast sarcomas [5]. We report a rare and first case of primary undifferentiated pleomorphic sarcoma of the breast diagnosed in a young patient after extensive tissue processing and immunohistochemistry.

## Case presentation

A 29-year-old female presented with a lump in the left breast for 2 months. She had no family history of any malignancy including the breast. On physical examination, the patient had a poorly demarcated mobile firm lump in the left breast. The lump was non-tender measuring 7 × 4 × 3 cm in the subareolar region. There was no evidence of axillary lymphadenopathy. The right breast appeared normal. The patient underwent total mastectomy without axillary dissection following core biopsy report of malignant spindle cell tumor.

**Fig. 1:**
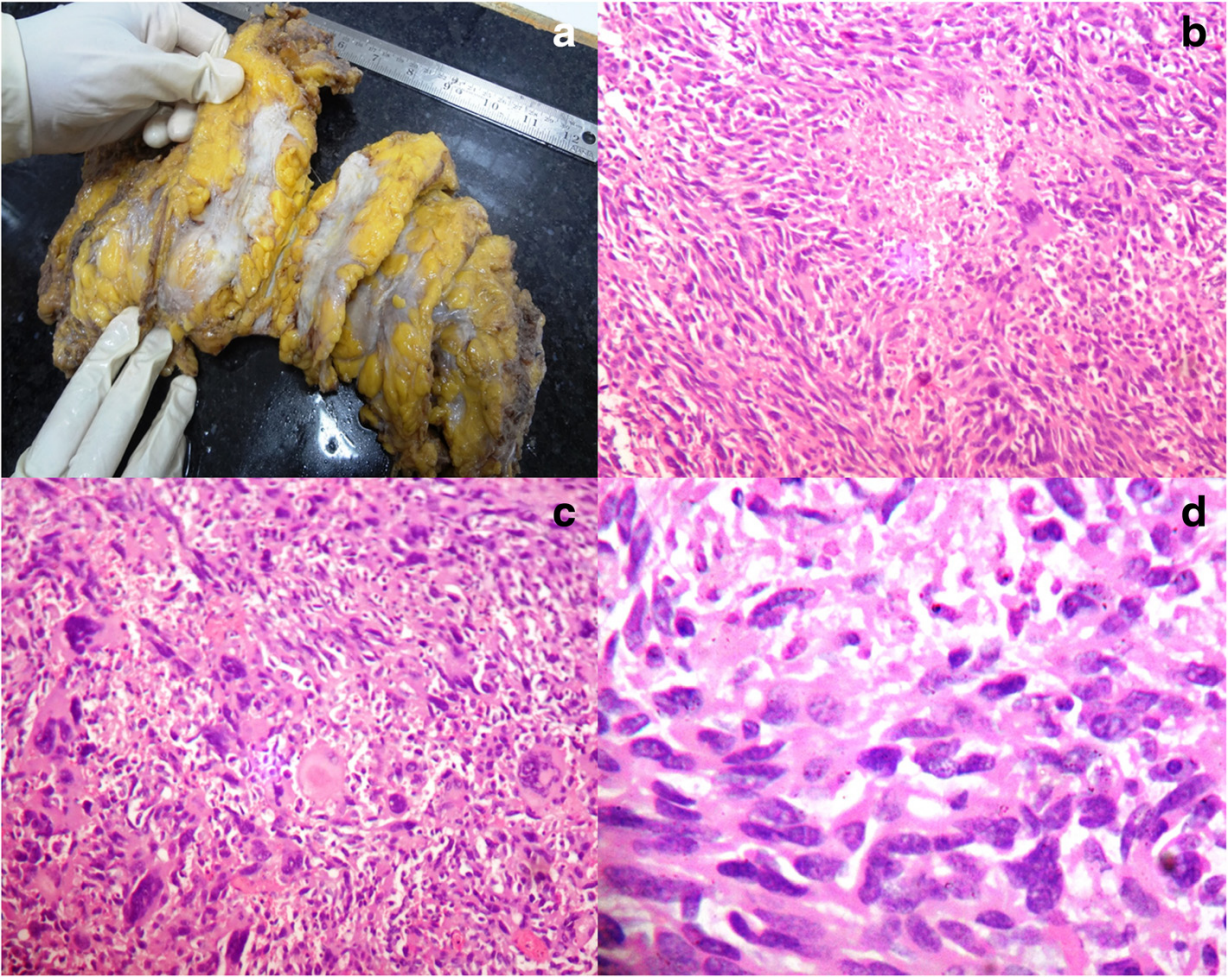
Mastectomy specimen showing fleshy ill-circumscribed lesion (**a**). Microphotograph showing the area of geographic necrosis surrounded by pleomorphic spindle cells (**b** H&E, 10×). Microphotograph showing osteoclast-like giant cells and atypical mitosis (**c** H&E, 10×). Microphotograph showing pleomorphic spindle cells and few inflammatory infiltrates (**d** H&E, 40×)

Gross examination of the mastectomy specimen revealed fleshy ill-circumscribed lesion (Fig. 1a). Thorough sampling of the lesion was done. Microscopic examination of the sections showed fascicles of spindle cells with hyperchromatic nuclei and eosinophilic cytoplasm tapering at the end intersecting with each other in a diffuse pattern. These spindle cells were seen palisading around the areas of geographic necrosis. Bizarre cells, multinucleated giant cells, and lymphocytes were seen admixed with the spindle cells. Mitotic count was >10/10 hpf (Fig. 1b–d). Focally adipose tissue was infiltrated by tumor cells. Initial diagnosis of spindle cell sarcoma was made. The morphological differential diagnosis at this stage was metaplastic carcinoma, leiomyoma, malignant peripheral nerve sheath tumor, malignant phyllode tumor, and stromal sarcoma.

**Fig. 2:**
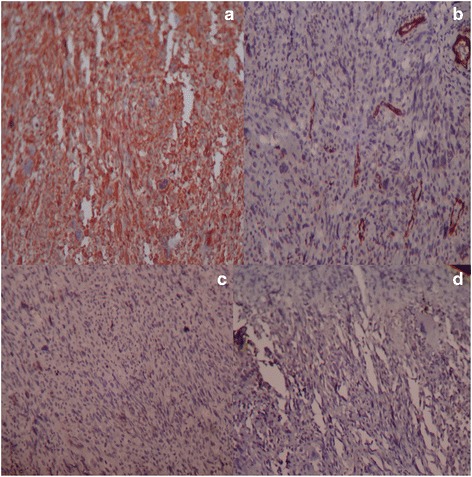
Undifferentiated pleomorphic sarcoma of the breast showing vimentin positivity (**a** IHC, 10×). Negative for smooth muscle actin (**b** IHC, 10×), S-100 protein (**c** IHC, 10×), and cytokeratin (**d** IHC, 10×)

On immunohistochemistry, only vimentin was positive. Cytokeratin (CK) and epithelial membrane antigen (EMA) negativity ruled out metaplastic carcinoma and phyllodes. Smooth muscle actin (SMA), desmin, CD64, and CD34 excluded leiomyoma, stromal sarcoma, inflammatory myofibroblastic tumor, and phyllode tumor. The possibility of malignant peripheral nerve sheath tumor and liposarcoma was not considered as S-100 protein and synaptophysin were negative (Fig. 2a–d). Based on histological features and immunohistochemical study, diagnosis of undifferentiated pleomorphic sarcoma was made.

### Discussion

Previous reports in literature suggest that 10.5–24 % of all primary breast sarcomas are undifferentiated pleomorphic sarcoma. It has been a diagnosis of exclusion following thorough sampling and critical use of ancillary diagnostic techniques [3, 6]. Most undifferentiated pleomorphic sarcomas of the breast affect mainly women over 64 years of age. To our knowledge, ours is the first case report in young women, but it has also been described in older women and men [7]. On gross examination, it can be identified as pale fibrous and fleshy areas admixed with zones of necrosis, hemorrhage, or myxoid features [8].

Microscopically, lesions exhibit cells showing marked pleomorphism admixed with bizarre giant cells, spindle cells, and variable foamy cells [9]. A storiform growth pattern and variable chronic inflammatory cells are also common [8]. However, clinical history, physical findings, or any histologic pattern is not specific of this tumor. We found many osteoclast-like giant cells also reported by Balbi et al. in literature, and it can lead to diagnostic difficulty mimicking malignant fibrous histiocytoma [10, 11]. Microscopically, it is impossible to differentiate as it mimics many of the malignant epithelial and mesenchymal tumors. Immunohistochemistry will be useful to distinguish primary breast sarcomas from non-mesenchymal malignant tumors and to delineate the level of differentiation of primary breast sarcomas [10, 12]. In our case, the patient underwent total mastectomy without axillary dissection, since these tumors rarely spread through the lymphatic system. The role of adjuvant chemotherapy and radiation also has been unclear [13]. Limited data in literature on undifferentiated pleomorphic sarcoma suggest an aggressive clinical course and high incidence of recurrence and metastasis [8].

## Conclusions

Undifferentiated pleomorphic sarcoma of the breast is a rare subtype of breast sarcoma as most of the breast cancers arise from epithelium. Presentation at a young age has not been reported in the literature till date, and our case presented at the age of 29 years. Initially, we had diagnostic difficulties to classify the lesion.

Based on histological findings and immunohistochemistry, we could diagnose the lesion. We suggest core biopsy with immunostaining is possibly superior to fine needle aspiration (FNA) as an initial diagnostic modality for breast masses with atypical features.

## Abbreviations

FNA, fine needle aspiration
